# Complementary roles of Pif1 helicase and single stranded DNA binding proteins in stimulating DNA replication through G-quadruplexes

**DOI:** 10.1093/nar/gkz608

**Published:** 2019-07-24

**Authors:** Melanie A Sparks, Saurabh P Singh, Peter M Burgers, Roberto Galletto

**Affiliations:** Department of Biochemistry and Molecular Biophysics, Washington University School of Medicine, Saint Louis, MO 63110, USA

## Abstract

G-quadruplexes (G4s) are stable secondary structures that can lead to the stalling of replication forks and cause genomic instability. Pif1 is a 5′ to 3′ helicase, localized to both the mitochondria and nucleus that can unwind G4s *in vitro* and prevent fork stalling at G4 forming sequences *in vivo*. Using *in vitro* primer extension assays, we show that both G4s and stable hairpins form barriers to nuclear and mitochondrial DNA polymerases δ and γ, respectively. However, while single-stranded DNA binding proteins (SSBs) readily promote replication through hairpins, SSBs are only effective in promoting replication through weak G4s. Using a series of G4s with increasing stabilities, we reveal a threshold above which G4 through-replication is inhibited even with SSBs present, and Pif1 helicase is required. Because Pif1 moves along the template strand with a 5′-3′-directionality, head-on collisions between Pif1 and polymerase δ or γ result in the stimulation of their 3′-exonuclease activity. Both nuclear RPA and mitochondrial SSB play a protective role during DNA replication by preventing excessive DNA degradation caused by the helicase-polymerase conflict.

## INTRODUCTION

During DNA replication, efficient progression of DNA synthesis can be hindered by obstacles derived from both exogenous and endogenous sources, such as DNA damaging agents, tightly binding proteins, and stable DNA secondary structures (1). Of the latter, G-quadruplexes formed by non-Watson–Crick base pairing of stretches of single-stranded DNA containing four to five tracks of guanines can pose a significant obstacle to DNA replication (2–9). This may occur especially during lagging-strand DNA synthesis when significant amounts of ssDNA are exposed that can form G-quadruplexes. Genome-wide analysis of yeast nuclear DNA has identified hundreds of sequences with G4-forming propensity (10), and thousands have been identified in the human genome (11). In yeast, timely and efficient DNA replication at G-quadruplex forming sequences requires the activity of the Pif1 family of helicases. In *Saccharomyces cerevisiae*, the activity of Pif1, the founding member of the Pif1 family of SF1B helicases (12,13), aids in DNA replication at hundreds of G-quadruplex motifs (14–16). In *Schizosaccharomyces pombe*, the orthologous helicase Pfh1 has similar functions at G-quadruplexes (17).

Both Pif1 and Pfh1 can unwind G-quadruplex structures *in vitro* (18–23). Indeed, Pif1 unwinds G-quadruplexes of different stability, albeit at different rates (18,21) and this activity is sufficient to allow DNA synthesis through a strong G-quadruplex (14). *In vivo*, the activity of Pif1 is required for timely DNA replication through strong G-quadruplexes on the lagging strand, while a weak G-quadruplex did not affect the overall progression of DNA replication (14). These observations raise the question whether only a subset of G4-forming sequences could indeed pose a significant problem to DNA replication and whether there is a threshold of G-quadruplex stability beyond which the activity of a non-replicative helicase is needed to aid the replication machinery. Below this threshold, perhaps the strand-displacement DNA synthesis activity of the lagging strand DNA polymerase δ (Pol δ) is sufficient to allow replication through marginally stable G-quadruplexes. Alternatively, formation of these structures may be prevented by binding of the single-stranded DNA binding protein RPA (24) or, if formed, they could be melted by RPA, thereby allowing unimpeded DNA synthesis. Indeed, RPA can melt some G-quadruplex structures *in vitro* (25,26).

The presence of G4 forming sequences is not restricted to nuclear DNA. Human mtDNA with its highly skewed GC content contains a large number of sequences predicted to form G-quadruplexes, and sites of mtDNA deletion breakpoints map in their proximity (27). Interestingly, analysis of the yeast mtDNA showed that, compared to nuclear DNA, it has a 10-fold higher frequency per kilo-base of G4-forming sequences (10), suggesting that G-quadruplexes may pose a problem during replication of mtDNA as well. However, it is possible that because the *S. cerevisiae* mitochondrial DNA polymerase δ Mip1 has significant stand displacement DNA synthesis activity (28), it may be sufficient to allow synthesis through G-quadruplexes. While Pif1 can be found in the nucleus and in mitochondria in both yeast and humans (29,30), the single-stranded DNA binding activity that could possibly destabilize secondary structures in the mitochondrial genome is provided by homo-oligomeric mtSSBs.

In this work, we tested a collection of G-quadruplexes with different stabilities as potential blocks for DNA synthesis by Pol δ in order to determine when the assisting activity of RPA and/or Pif1 is required for efficient synthesis through these blocks. We also show that G-quadruplexes may pose a problem for DNA replication in mitochondria and similarly address the roles of mtSSB and Pif1 in replication through blocks by Pol δ. Finally, in performing these studies we discovered a novel and unexpected function of single-stranded DNA binding proteins in preventing head-on conflicts between the polymerase and the helicase that would otherwise result in excessive DNA degradation by the polymerase proofreading activity.

## MATERIALS AND METHODS

### Proteins and DNA substrates

Both variants of *S. cerevisiae* DNA polymerase δ, Pol δ and Pol δ^DV^ (D520V), were purified from a yeast overexpression system as described (31). *Saccharomyces cerevisiae* RPA, PCNA, and RFC were purified as previously described (32–34). *Saccharomyces cerevisiae* Pif1 was purified as previously described (35). The coding sequence for full-length *S. pombe* Pfh1 was synthesized by GenScript (Piscataway, USA) codon-optimized for overexpression in *Escherichia coli* and cloned in pET28b at NdeI/XhoI, leaving a N-terminus His_6_. Pfh1 was purified following the same protocol as for Pif1 (35). The purification of *S. cerevisiae* Mip1 from E. coli is detailed in Supporting Information. DNA substrates were purchased from Integrated DNA Technologies (Coralville, IA) and their sequences are listed in Supplementary Table S1. The 5′-fluorescently labeled primer was annealed to a 3′-biotinylated DNA strand in the presence of 20 mM Tris, pH 8.0, 8 mM MgCl_2_ and 150 mM KCl. The reaction was heated to 95°C and allowed to slowly reach room temperature. G quadruplex formation occurred naturally under the annealing conditions.

### G-quadruplex formation and stability

G4s were formed by adding the G4 containing oligonucleotide to the specified buffer at 3–4 μM concentration for spectroscopic assays and at 1 μM for the replication assays, heating the solution to 95°C in a hot water bath, and allowing it to equilibrate to room temperature overnight. The structure of the formed G4s was examined by circular dichroism (CD) spectroscopy (Jasco, J-715). UV melting assays were carried out to measure G4 stability and were performed as previously described (14). Briefly, G4s were assayed by monitoring the change in absorbance at 295 nm on a Varian Cary-100 spectrophotometer equipped with a Peltier-controlled cuvette holder. After incubating the G4 containing oligonucleotide for 10 min at the starting temperature (14–16°C), the temperature was raised by 2°C increments and absorbance was measured after 3 min incubation at each temperature. Normalized change in absorbance at 295 nm was fitted with a two-state model using GraphPad Prism.

### Replication assay

Replication assays were carried out in Buffer TM (20 mM Tris–HCl pH 7.8, 8 mM MgAc_2_, 1 mM DTT, 0.1 mg/ml BSA) with 100 mM KCl. Replication assays were performed with 20 nM of the DNA substrates, a concentration at which the G-quadruplexes, containing four G-tracks, are predominantly unimolecular (Supplementary Figure S1A) (4,18,21,36,37). For experiments with Pol δ, a standard loading protocol was followed (35). The concentrations reported are the final ones after starting the reaction. RFC (20 nM) and PCNA (20 nM) were allowed to react with a single biotinylated DNA substrate (20 nM) in the presence of streptavidin (20 nM) and ATP (1 mM) for 2 min at 30°C, followed by the addition of Pol δ (20 nM) and dNTP mix (100 μM). When mentioned, RPA (80 nM) was added before Pol δ and incubated for 30 s at 30°C. Pif1 (40 nM) was added with Pol δ. The experiments with Mip1 were performed similarly but lacked ATP, PCNA, RFC and streptavidin. At the indicated times the reactions were stopped by the addition of 80 mM EDTA, 0.08% SDS and incubated at 55°C for 10 min. After addition of formamide (50% final) and bromophenol blue, the samples were heated at 95°C for 2 min and analyzed on a 12% denaturing polyacrylamide gel, pre-run for 2 h in 1× TBE. The gels were scanned using a Typhoon 9400 Variable Mode Imager (GE Healthcare), monitoring the Cy3 fluorescence of the labeled primer. Accumulation of full-length product was quantified using ImageQuant; the background was subtracted using the rubber-band option in ImageQuant and the intensity of the full-length product was normalized to the intensity of the whole lane. The reported values in the figures are the mean and standard deviation from three to five independent replicates. For the statistical significance of the difference between data a t-test was performed with GraphPad Prism.

## RESULTS

### hTelo and cMyc G-quadruplexes block DNA synthesis by DNA polymerase δ

Two well-studied G-quadruplexes (G4s) are formed by four TTAGGG repeats found at human telomeres (hTelo) and at the promoter region of the human c-Myc proto-oncogene (cMyc). In this study, we used the Pu27 sequence found in the c-Myc promoter, containing five G-tracks that can form two G-quadruplexes (38,39), both in parallel orientation (18,21,40). On the other hand, hTelo contains four G-tracks that form a single antiparallel structure (18,21,36). G-quadruplex formation and thermal stability were examined by CD spectroscopy and UV melting. In the buffer conditions used for DNA replication assays, both cMyc and hTelo adopt the expected configurations (Supplementary Figure S1B). The melting temperature of hTelo was dependent on the K^+^ concentration (Supplementary Figure S1C), with the addition of Mg^2+^ providing further stabilization (Supplementary Figure S1D), as expected (22). However, cMyc forms a more stable structure, as melting could only be observed at low KCl concentrations and the addition of Mg^2+^ was enough to stabilize cMyc to an extent that prevented melting (Supplementary Figure S1E). Thus, cMyc forms a more stable G-quadruplex than hTelo.

**Figure 1.**
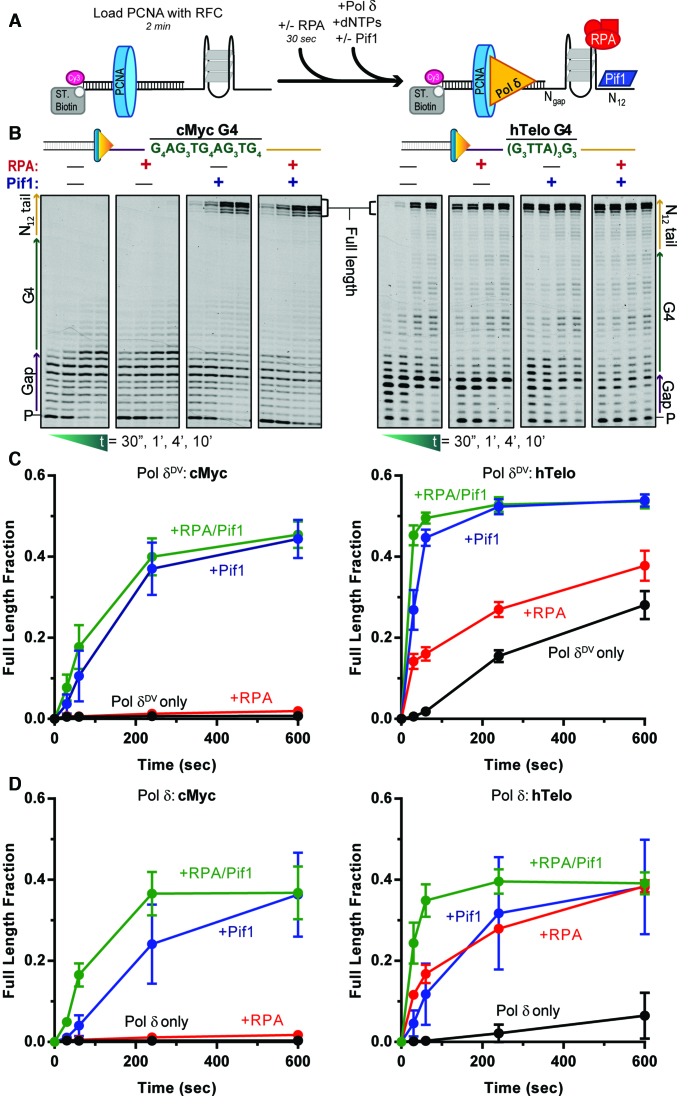
Pif1, but not RPA, aids Pol δ in DNA synthesis through hTelo and cMyc G-quadruplexes. (**A**) Schematic of the primer extension reaction scheme. (**B**) Representative primer extension assay results for Pol δ^DV^ alone (black) and in the presence of either RPA (red) or Pif1 (blue) or both (green), with cMyc (left) or hTelo (right) in the template strand. Quantifications of the fraction of full-length product generated as a function of time are shown in (**C**) for Pol δ^DV^ and (**D**) for Pol δ.

Next, we used primer extension assays to test the ability of cMyc and hTelo to affect DNA synthesis by DNA polymerase δ (Pol δ). Figure 1A shows a schematic of the reaction. Briefly, a primer labeled at its 5′ end with Cy3 is annealed to a template that contains: (i) a biotin at its 3′ end to allow binding of streptavidin and prevent PCNA from sliding off the DNA; (ii) a G4-forming sequence at variable distances from the 3′ terminus of the primer (gap) and (iii) a 12 nt 5′ extension as an entry point for the 5′ to 3′ helicase. Primer extension assays were performed with both an exonuclease-deficient variant (Pol δ^DV^, D520V) (Figure 1B and C) and wild type Pol δ (Figure 1D and Supplementary Figure S2). Pol δ^DV^ was used to prevent exonucleolytic digestion of primer; moreover, because of the lack of exonuclease activity, it has stronger strand displacement DNA synthesis activity (41). Consistent with cMyc forming a highly stable G4 structure, its presence on the template strand prevented any formation of full-length product by Pol δ^DV^ (Figure 1B and C), with most of the DNA synthesis halted in the gap region of the template just before the start of cMyc. The same is true for Pol δ (Figure 1D and Supplementary Figure S2). Comparably, for the less stable hTelo at 10 min no >30% of full-length products were generated by Pol δ^DV^ (Figure 1C) and <10% by Pol δ (Figure 1D). Furthermore, G4 stability can be altered by the use of Na^+^, that does not stabilize G4s as well as K^+^ (21). In the presence of NaCl (Supplementary Figure S3A), DNA synthesis of an unstructured G-rich single stranded DNA template is unaffected, as expected, while the extent of DNA synthesis through a G4 was increased, consistent with replicating through a weaker G4. These results indicate that the stability of the formed G-quadruplexes correlates with their blocking strength during DNA synthesis and that the polymerization activity of Pol δ may not be enough to fully destabilize them, requiring additional factors. Therefore, we tested the role of RPA, that can melt DNA secondary structures including G-quadruplexes (25,42,43), and Pif1, that unwinds G-quadruplexes (19,21,22), in aiding Pol δ in DNA synthesis through cMyc and hTelo.

Consistent with previous results using G4-forming sequences found in the yeast genome (14), in the presence of Pif1, full length DNA synthesis products were formed for both cMyc and hTelo, independent of whether Pol δ^DV^ or Pol δ were used (Figure 1 and Supplementary Figure S2). Stimulation of DNA synthesis through cMyc and hTelo strictly depended on the ATP-dependent DNA unwinding activity of the helicase core of Pif1, as an ATPase dead mutant was unable to stimulate DNA synthesis even when present in large excess relative to the DNA (Supplementary Figure S3B). Interestingly, in experiments with Pol δ, the addition of Pif1 appears to increase primer degradation, and this increase is suppressed when RPA is added concurrently (Supplementary Figure S2, green box). This observation will be further elaborated on in the last section of the Results.

Surprisingly, when using either Pol δ or Pol δ^DV^, RPA was unable to aid DNA synthesis through cMyc (Figure 1), even with a longer ssDNA gap (Supplementary Figure S3C) or in the presence of large excess of RPA relative to DNA (tested on similarly strong G4 in Supplementary Figure S7). On the other hand, RPA provides significant aid in DNA synthesis through the weaker hTelo, especially for wild-type Pol δ that has a weaker intrinsic strand displacement DNA synthesis activity than the exonuclease-defective form (Figure 1 and Supplementary Figure S2). Furthermore, for Pol δ when RPA and Pif1 are present at the same time, both destabilization by RPA and unwinding by Pif1 of the G-quadruplex structures contribute to an increase in the rate of DNA synthesis (Figure 1D).

### G-quadruplex stability correlates with blocking strength and the ability of Pif1, but not RPA, to aid in DNA synthesis

The experiments in the previous section were performed with hTelo that has an average G track and loop length of mixed sequence of 3 nt (G_3_N_3_) and cMyc that has an average G track length of 3.6 nt and loop length of just 1 nt (G_3.6_N_1_). Changes in the number of G stacks or the loop length have been shown to modulate the stability of G-quadruplexes and affect the structure of the G4 (44–46), providing a tool to test how their stability and structure (e.g. parallel versus anti-parallel, number of G-tracks, loop length) correlate with their strength in blocking DNA synthesis, and how RPA and Pif1 may stimulate DNA synthesis through these blocks. To this end, we designed a series of artificial G-quadruplexes in which the number of stacked Gs and the loop length were varied (Supplementary Table S1). To avoid potential sequence effects from the loops, each loop contained only thymidines (T_*x*_). CD spectroscopy and UV melting experiments confirmed that for the (G_3_T_*x*_)_3_G_3_ sequences, with three stacked Gs, decreasing the number of Ts in the loop increases the stability of the G-quadruplex and increases their propensity to form parallel structures (Supplementary Figure S4A). However, independent of the loop length, the (G_4_T_*x*_)_3_G_4_ sequences with four stacked Gs form parallel and highly stable G-quadruplexes (Supplementary Figure S4B).

With the (G_3_T_*x*_)_3_G_3_ sequences in the template strand, primer extension assays show that the fraction of full-length DNA synthesis products generated by Pol δ decreases as the loop length decreases (Supplementary Figure S4C), paralleling the increase in stability of the G4. As expected, Pol δ^DV^ was slightly better than wild-type in DNA synthesis through the weaker (G_3_T_*x*_)_3_G_3_ sequences, but no full-length products were generated with a loop size of 1 nt. In stark contrast, with the (G_4_T_*x*_)_3_G_4_ sequences no full-length products were generated by either Pol δ or Pol δ^DV^, independent of the loop length.

**Figure 2.**
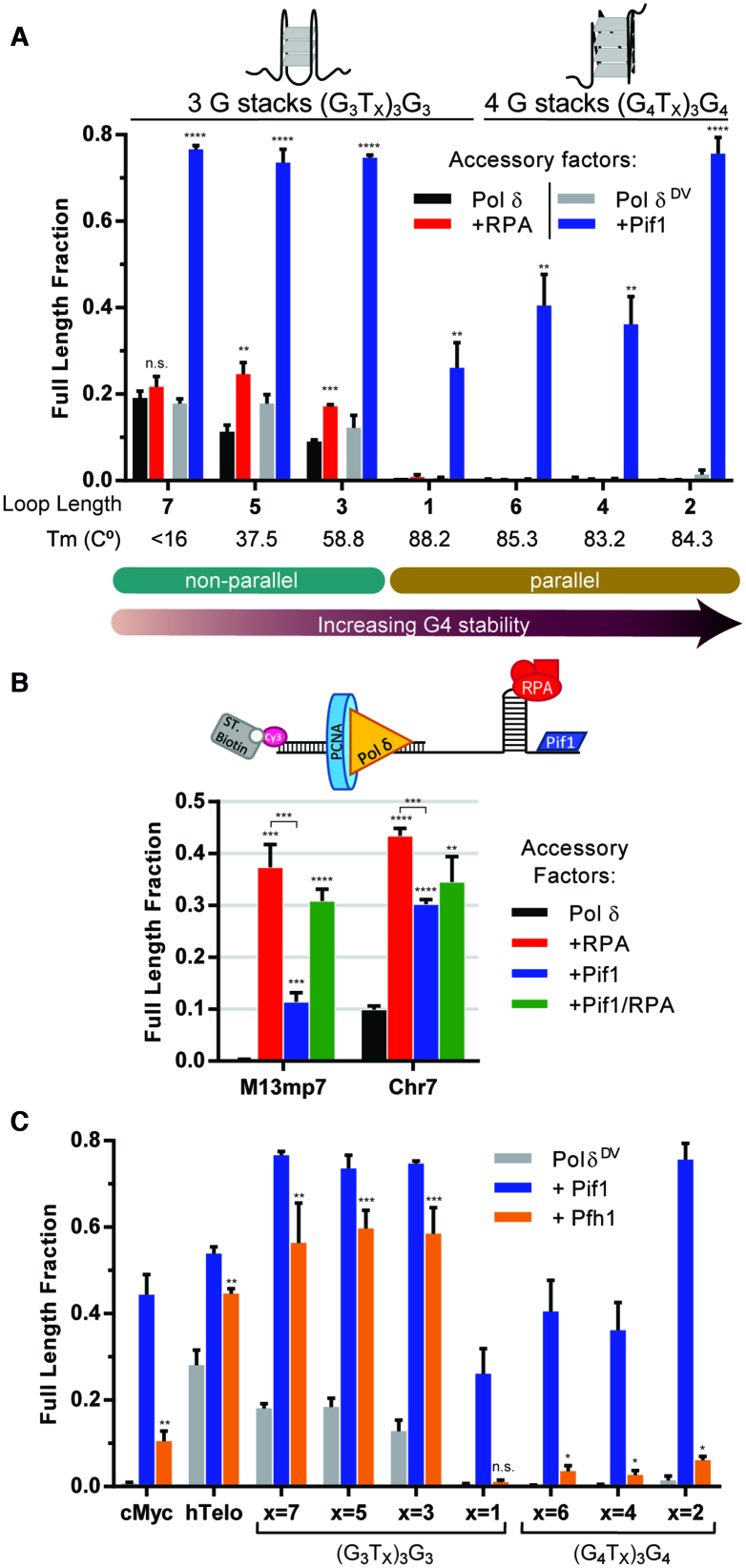
DNA synthesis by Pol δ through model G-quadruplexes of different stability and DNA hairpins. (**A**) Quantification of the fraction of full-length product, for G4s that differ in the number of G in the stack and loop length, generated by either Pol δ with (red) and without RPA (black) or Pol δ^DV^ with (blue) and without Pif1 (gray) at the 10 min time point. Non-parallel configurations include antiparallel and mixed antiparallel and hybrids conformations. Melting temperatures are indicated below each G4 for G4s formed in 30 mM KCl and 8 mM MgAc_2_. (G_4_T_*x*_)_3_G_4_ and (G_3_T_1_)_3_G_3_ form parallel structures, while (G_3_T_3–7_)_3_G_3_ form nonparallel structures. (**B**) Quantification of the fraction of full-length product generated by Pol δ (black) for two hairpins (M13mp7 or Chr7) in the presence of RPA (red), Pif1 (blue) or both (green). (**C**) Fraction of full-length product generated at the 10 min time point, for the indicated G4s, comparing the activity of Pif1 (blue) and Pfh1 (orange).

The role of Pif1 in aiding in DNA synthesis through (G_3_T_*x*_)_3_G_3_ and (G_4_T_*x*_)_3_G_4_ sequences was tested using Pol δ^DV^ in order to avoid the substantial Pif1-stimulated degradation of the primer by the 3′-exonuclease activity of Pol δ (see below). Representative primer extension assays for each G4 sequence tested are shown in Supplementary Figure S5. Remarkably, Pif1 was able to aid in DNA synthesis regardless of the number of stacked Gs or loop length (Figure 2A), consistent with the helicase activity being sufficient to unwind these structures independent of their stability. Moreover, Pif1 requires a 5′-ssDNA entry point for its motor protein activity (22), and in the absence of this entry point loops of 6 or 12 nt within the G4 structure are not sufficient for significant Pif1-dependent stimulation of DNA synthesis (Supplementary Figure S5C).

In order to amplify any potential effect of RPA, its role in DNA synthesis through the same G4 sequences was tested using Pol δ (Figure 2A and Supplementary Figure S6). In stark contrast to Pif1, RPA only partially aided DNA synthesis and only for the weaker (G_3_T_*x*_)_3_G_3_ sequences. No full-length product was detected with (G_3_T_1_)_3_G_3_ or any of the (G_4_T_*x*_)_3_G_4_ sequences. For the (G_3_T_*x*_)_3_G_3_ sequences, our results show an inverse correlation between the increase in G4 stability at shorter loop lengths and the ability of yeast RPA to aid in DNA synthesis. These results agree with a previous study by Ray *et. al.* (45), where the ability of human RPA to melt G-quadruplexes was directly correlated to the length of the loop and inversely correlated to the number of stacked Gs. Thus, one would predict a significant effect of RPA in aiding DNA synthesis through the (G_4_T_x_)_3_G_4_ sequences with long loops. However, our results show no effect of RPA with any of the (G_4_T_*x*_)_3_G_4_ sequences despite a loop length of even 12 nt and a 5-fold higher RPA concentration (Supplementary Figure S7). These results suggest the presence of a threshold in the strength of the G-quadruplexes above which DNA synthesis by Pol δ is completely halted even in the presence of RPA, thereby requiring the helicase activity of Pif1 to unwind the G4 structure and relieve the block to DNA replication.

To determine if this threshold in blocking strength was specific to G4s, next we tested the effect of two stable hairpins on Pol δ DNA synthesis: the hairpin from the MCS of M13mp7 (47) and Chr7hp, a naturally occurring hairpin found in chromosome 7 of *S. cerevisiae*. As seen in Figure 2B and Supplementary Figure S8, both hairpins are strong blocks that either completely (M13mp7) or significantly (Chr7) block Pol δ. Interesting, and in contrast to G4s, the addition of RPA is sufficient to allow replication through both of these strong hairpins. While Pif1 facilitates DNA synthesis through the hairpins, its addition also stimulates DNA degradation by Pol δ, which is prevented by the presence of RPA (this phenomenon will be further discussed later). These results suggest that the need for Pif1 in getting through DNA secondary structures is unique to G4s, as RPA is sufficient to allow replication through a strong hairpin.

Finally, using the heterologous *S. cerevisiae* Pol δ as an example of a general polymerase we tested the activity of *S. pombe* Pfh1 helicase in helping DNA synthesis through G-quadruplexes. Pfh1 is a member of the conserved Pif1-family and also another example of a 5′-3′ helicase. Figure 2C summarizes the results of primer extension assays performed with different G-quadruplexes. Pfh1 allows DNA synthesis through the weaker G-quadruplexes to an extent similar to Pif1. These data suggest that the ability to facilitate DNA synthesis through G-quadruplexes is a conserved property of the Pif1-family of helicases, and possibly a general feature of 5′-3′ helicases. However, this activity will also depend on the intrinsic G-quadruplex unwinding activity of the specific helicase. Indeed, Pfh1 has a limited effect on DNA synthesis through the strong G-quadruplexes, suggesting that, compared to Pif1, Pfh1 has weaker G-quadruplex unwinding activity.

### Pif1 aids DNA synthesis by the mitochondrial DNA polymerase through G4s

Analysis of mtDNA predicts a higher density of G4-forming motifs compared to the nuclear genome (10), suggesting that G quadruplexes may pose a challenge to mitochondrial replication as well. Thus, we tested whether G-quadruplexes would hinder the DNA synthesis activity of the *S. cerevisiae* mitochondrial DNA polymerase γ, Mip1 and the role that Pif1 and mtSSBs may play in the process.

**Figure 3.**
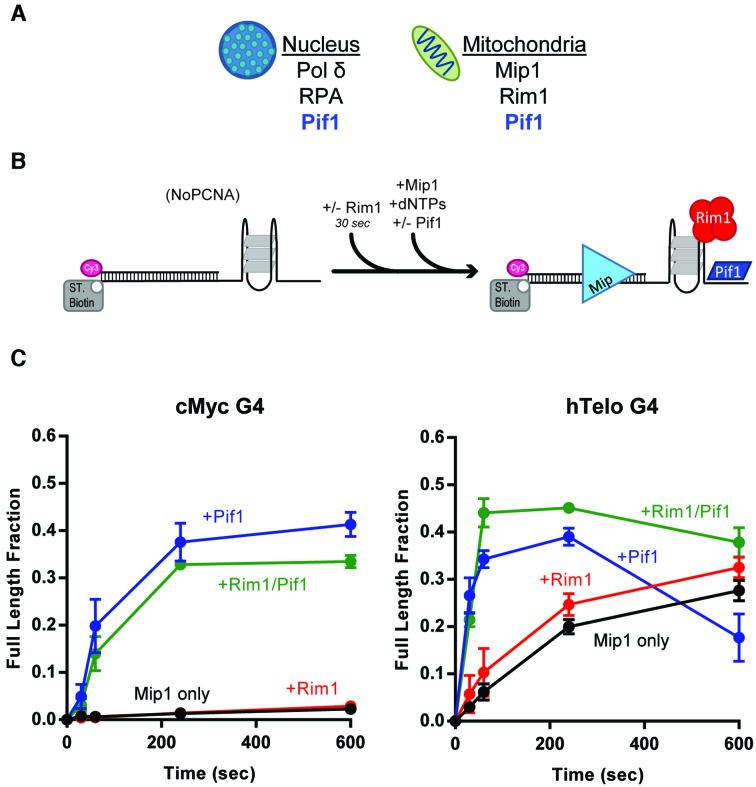
Pif1, but not mtSSBs, aids Mip1 in DNA synthesis by through G-quadruplexes. (**A**) Cartoon showing the different polymerases and single-stranded DNA binding proteins found in the nucleus and mitochondria in yeast. (**B**) Schematic of the primer extension reaction scheme. (**C**) Quantification of the fraction of full-length product generated by Mip1 alone (black) or in the presence of either ScRim1 (red) or Pif1 (blue) or both (green), with cMyc (left) and hTelo (right) in the template strand.

We overexpressed and purified Mip1 that is active in strand displacement DNA synthesis and has a strong 3′-5′ exonuclease activity (Supplementary Figure S9). Representative primer extension assays with either cMyc or hTelo in the template strand are shown in Supplementary Figure S10. No full-length DNA synthesis products were detected with cMyc, while no more than 30% of full-length products are generated with hTelo (Figure 3C). This blocking ability is unique to G4 structures, as Mip1 was able to carry out DNA synthesis through DNA hairpins (Supplementary Figure S11). Thus, similar to what was observed with Pol δ, a G-quadruplex is a barrier to DNA synthesis by a mitochondrial polymerase and the extent of synthesis inversely correlates with the strength of the formed G-quadruplex.

Next, we tested the ability of Rim1 to aid in DNA synthesis by Mip1. Because the *S. cerevisiae* mitochondrial SSB, Rim1 can form either dimers or tetramers in solution, with tetramers being favored on ssDNA (48), primer extension assays were performed in a large excess of Rim1 over the DNA concentration to promote tetramer formation. However, Rim1 did not significantly stimulate DNA synthesis through the G-quadruplex blocks (Figure 3C), and the same is the case for human mtSSB, which forms stable tetramers (Supplementary Figure S10B). On the other hand, the presence of Pif1 promoted significant DNA synthesis by Mip1, independent of the strength of the G-quadruplex. Thus, unwinding of the G-quadruplex by Pif1 is required for efficient DNA synthesis by the mitochondrial DNA polymerase as well. Again, we note that, like with Pol δ, Pif1 appears to stimulate primer degradation by the exonuclease activity of Mip1 (Supplementary Figure S11, green boxes). For example, with the weaker hTelo, the increase in exonuclease activity leads to an apparent decrease in full-length products generated at the longer times, that can be suppressed by the presence of Rim1 (Figure 3C).

### Helicase–polymerase conflicts lead to stimulation of exonuclease activity and are suppressed by single stranded DNA binding proteins

**Figure 4.**
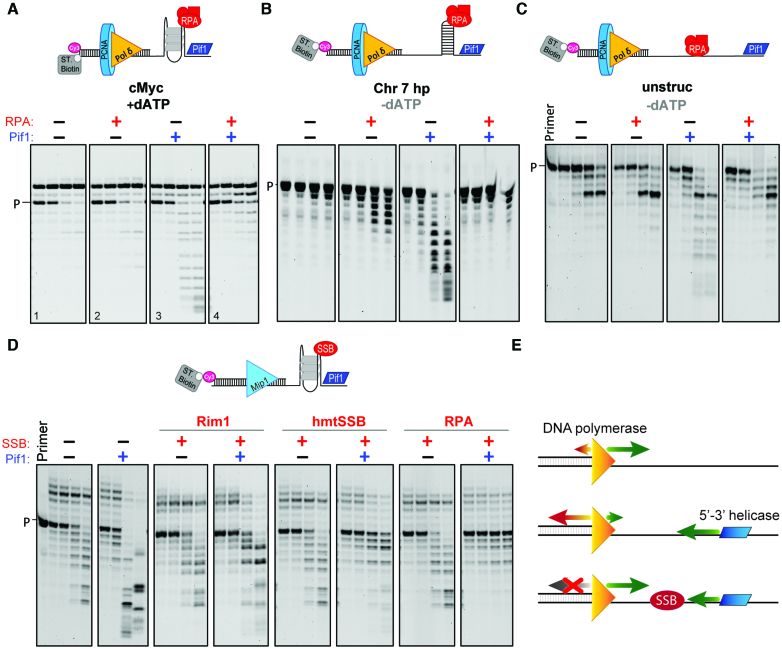
Head-on polymerase-helicase conflicts result in overt exonuclease activity. (**A**) Exonuclease digestion of the primer, under conditions of limited DNA synthesis (dATP only), by Pol δ alone (panel 1) or in the presence of either RPA (panel 2) or Pif1 (panel 3) or both (panel 4). The substrate contains cMyc in the template placed 20 nt away from the 3′ end of the primer. (**B** and **C**) The same assays but in the absence of DNA synthesis and using DNA substrates that contain either a hairpin sequence or no DNA secondary structures in the template. (**D**) Exonuclease digestion of the primer by Mip1 alone or in the presence of the indicated combinations of Pif1, ScRim1, human mtSSB, or RPA. (**E**) Model of helicase-polymerase conflict. In the absence of a 5′-3′ helicase, DNA polymerases favor DNA synthesis over DNA degradation. However, head-conflict between the translocating helicase and the polymerase bound at the 3′-end of the primer results in stimulation of its exonuclease activity. The presence of a single-stranded DNA binding protein (SSB) protects against the helicase-polymerase conflict. Quantification of these gels are shown in Supplementary Figure S14.

The increase in primer degradation by Pol δ and Mip1 when Pif1 was added in the absence of a single-stranded DNA binding protein was unexpected and examined further. Primer degradation is due to the exonuclease activity of the polymerases, as no primer degradation was observed with Pol δ^DV^ or when Pif1 was added to the substrate in the absence of the polymerase (Supplementary Figure S13A). Next, we performed primer extension assays under conditions of limited DNA synthesis (only dATP added) and substrates with cMyc placed 20 nt (Figure 4A) or 8 nt (Supplementary Figure S12A) from the 3′-end of the primer. Independent of the gap length or the presence of RPA, Pol δ incorporates two or four As, with little degradation occurring at the long time points. However, addition of Pif1 leads to degradation of the newly extended primer, and this degradation can be suppressed by the concurrent addition of RPA. The same is true in the absence of DNA synthesis (Supplementary Figure S13D), in which case Pif1 can use the ATP from the PCNA-loading step for translocation and unwinding. In these reactions the streptavidin block was placed on the primer instead of the template strand to limit the exonuclease activity of PCNA-loaded Pol δ and allowed clearer observed stimulation of primer degradation by Pif1.

The observed Pif1-dependent stimulation of primer degradation does not require the presence of the specific structure formed by a G4, as the same phenomenon is observed with hairpins (Figure 4B, Supplementary Figure S12B) or a ssDNA template (Figure 4C). One possible mechanism that could explain the observed increase in primer degradation is that the helicase completely unwinds the dsDNA region, releasing the primer from the template, followed by the exonucleolytic digestion of the free single stranded primer by the polymerase. However, control experiments suggest that this is not the case. The degradation pattern of the ssDNA primer by Pol δ is different from the degradation pattern of the primer in experiments where Pif1 was pre-incubated with PCNA-loaded DNA before addition of the Pol δ (Supplementary Figure S13B and C).

Pif1-dependent increase in exonucleolytic digestion of the primer was also observed with the mitochondrial DNA polymerase Mip1 (Supplementary Figure S11A, green box). Thus, primer extension assays with Mip1 were repeated in the presence of only dATP to limit DNA synthesis. Because Mip1 has a strong intrinsic exonuclease activity these assays were performed under conditions that favor nucleotide incorporation and limit degradation: (i) high dATP concentration (1 mM); (ii) a DNA substrate with streptavidin bound to the primer and cMyc placed 8 nt from the primer junction and (iii) lower salt concentration (40 mM KCl). Under these conditions, the presence of Pif1 significantly increases the level of exonucleolytic digestion of the primer (Figure 4D). Similar to what observed with Pol δ, the Pif1-dependent stimulation of Mip1 exonuclease activity can be suppressed by the presence of single-stranded DNA binding proteins, albeit to a different extent.

## DISCUSSION

G-quadruplexes can pose a significant obstacle to DNA replication and multiple DNA polymerases have been shown to be stalled by the presence of a G-quadruplex formed on the template strand (2–9), including *S. cerevisiae* DNA polymerase δ (14). The question is how these blocks are dealt with to allow progression of DNA synthesis, the ultimate goal during DNA replication. Growing experimental evidence points to the activity of non-replicative helicases for efficient DNA replication through these structural blocks and genome maintenance (49–53). In *S. cerevisiae*, the activity of Pif1 is required for timely DNA replication at strong G-quadruplexes during lagging strand DNA synthesis, while a weak G-quadruplex did not affect DNA replication (14). Here, we sought to investigate if only a subset of G4-forming sequences would pose a significant problem to DNA replication by DNA polymerase δ and, thus, beyond a threshold of G-quadruplex stability the activity of a non-replicative helicase would be needed to aid the replication machinery. Furthermore, because RPA has been shown to be able to melt G-quadruplex structures, we sought to understand the role that RPA may play in aiding DNA synthesis through G-quadruplexes.

In *S. cerevisiae*, genome-wide analysis of all G4-forming sequences indicated an average of 4.3 G-tracts with an average length of 3.3 nt and an average loop length of 12.2 nt (10). Based on the work by Ray *et al.* (45) and our data here, G-quadruplexes with 3 nt in the G-tract and long loops may not form stable structures. The stability of the G-quadruplexes formed with the (G_3_T_*x*_)_3_G_3_ sequences decreases with increasing loop length and we would expect that with an average 12.2 nt loop length only marginally stable structures would form, thereby potentially posing less of a problem during DNA replication. Indeed, the DNA synthesis activity of Pol δ through these G-quadruplexes inversely correlates with their stability and RPA aids in the process, indicating that it can, at least in part, melt these weaker structures. Furthermore, RPA stimulates DNA synthesis by Pol δ through the human telomere G-quadruplex hTelo, which contains 4 G-tracts with a length of 3 nt and a loop of 3 nt. These results agree with published results showing that RPA can melt hTelo *in vitro* (25,42,43). However, a robust stimulation of DNA synthesis is observed only in the presence of Pif1, indicating that even for these weaker G-quadruplexes, the helicase activity of Pif1 is required for efficient DNA replication *in vitro*. This suggests that the limited DNA synthesis through these weaker G-quadruplexes observed in the absence of Pif1 may not be enough to sustain efficient DNA replication *in vivo*. Indeed, a weak G-quadruplex that *in vitro* could be replicated through by Pol δ and RPA alone, did not cause measurable stalling of the DNA replication fork in a yeast *pif1*Δ mutant, whereas a strong G-quadruplex did (14).

When genome-wide analysis of yeast G4 motifs was restricted to sequences that are conserved within the *Saccharomyces* genus, on average these sequence have 5.1 G-tracts with an average length of 3.7 nt and an average loop length of 10.8 nt (10). Similarly, the Pu27 sequence found in the promoter of *c-MYC* proto-oncogene has five G-tracts with an average length of 3.6 nt and short 1 nt loops. In general, increase in the length of the G-tract and decrease in loop length are expected to stabilize the formed G-quadruplexes (46). In this work, we showed that cMyc, the (G_3_T_1_)_3_G_3_ sequence and the (G_4_T_x_)_3_G_4_ sequences form highly stable structures that completely block DNA synthesis by Pol δ, with no significant stimulation of through-synthesis provided by RPA. Any significant DNA synthesis through these more stable G-quadruplexes requires the helicase activity of Pif1, consistent with our recent observations *in vivo* and *in vitro* using a strong G4-forming sequence naturally found in yeast (14). These results suggest that there may exist a threshold in the stability of G-quadruplexes beyond which the activity of non-replicative helicases is required for DNA replication. Members of the SF1B Pif1-family are conserved from bacteria to yeast and humans, and heterologous expression of different Pif1-family members can suppress gross-chromosomal rearrangements at G4 motifs in yeast (54), indicating that these helicases have similar functions at G-quadruplexes in these different organisms. Indeed, we showed here that, similar to Pif1, *S. pombe* Pfh1 can facilitate DNA synthesis through G-quadruplexes. However, the data also suggest that Pfh1 has a weaker G-quadruplex unwinding activity and that additional species-specific factors may be required to facilitate DNA synthesis through strong G-quadruplexes in *S. pombe*. Also, we note that because these helicases unwind with a 5′ to 3′ directionality, they can gain access to the ssDNA downstream of the G-quadruplex, which would be available during lagging strand DNA replication. This may not be the case for 3′ to 5′ helicases for which access to ssDNA may be precluded if DNA synthesis proceeds to the 3′ edge of the G-quadruplex.

The presence of G4 forming sequences is not restricted to nuclear DNA. Human mtDNA with its highly skewed GC content contains a large number of sequences predicted to form G-quadruplexes, and sites of mtDNA deletion breakpoint map in their proximity (27). Interestingly, analysis of the yeast mtDNA showed that, compared to nuclear DNA, it has a 10-fold higher frequency per kilo-base of G4-forming sequences, suggesting that G-quadruplexes may pose a problem also during replication of mtDNA. Consistent with this possibility, in this work we showed that the strong G-quadruplex formed by cMyc blocks DNA synthesis of the yeast mitochondrial DNA polymerase Mip1, with mtSSBs providing no significant stimulation. Pif1 substantially stimulates G-quadruplex through-synthesis by Mip1. Thus, the requirement of the helicase activity of Pif1 to facilitate DNA synthesis through G-quadruplexes is shared by the DNA replication machinery in the nucleus and in mitochondria. Moreover, the ability of Pif1 to stimulate DNA synthesis of Mip1 in mitochondria, which do not utilize PCNA, reinforces the proposal that the reported interaction between Pif1 and PCNA is important for localization of Pif1 at the site of action rather than for stimulation of its activity (14).

While the results presented in this work may argue that, compared to Pif1, RPA has a limited role in helping DNA replication across G-quadruplexes, this is not to say that neither RPA nor SSBs have a role in the process. Our results reveal a novel and general function of single-stranded DNA binding proteins in preventing head-on conflicts between the polymerase bound to the primer and the helicase. Unhindered translocation of Pif1 along the template strand elicits excessive exonuclease activity by the polymerase. The data suggests that this is not due to full unwinding of the primer by Pif1. Rather, upon reaching the 3′ ds/ss junction Pif1 may unwind part of the primer releasing its 3′-end and making it accessible to the exonucleolytic site on the polymerase. More intriguingly, Pif1 may push the polymerase in the direction opposite to DNA synthesis, thereby forcing the 3′-end into the exonucleolytic site. The available data do not allow us to unambiguously distinguish between these two possibilities. However, we note that Pif1 can push directionally a SSB tightly bound to ssDNA (55), and it can also displace proteins tightly bound to dsDNA (31), suggesting that the motor protein activity of Pif1 may be enough to act on the polymerase bound to the primer. In conclusion, we propose that during lagging strand replication Pif1 is required for unwinding of G-quadruplexes to allow efficient progression of DNA synthesis, while single-stranded DNA binding proteins generate a bumper between the polymerase and the incoming helicase, thereby preventing head-on collisions.

## Supplementary Material

gkz608_Supplemental_File
