# Supplementary material for: Complementary roles of Pif1 helicase and single stranded DNA binding proteins in stimulating DNA replication through G-quadruplexes
Source: Nucleic Acids Res. 2019 Jul 24;47(16):8595–605. doi: 10.1093/nar/gkz608 (PMC7145523; doi:10.1093/nar/gkz608)

Supporting Information

Complementary roles of Pif1 helicase and RPA in stimulating DNA replication through G-quadruplexes

Melanie A. Sparks, Saurabh P. Singh, Peter M. Burgers and Roberto Galletto[#]

Department of Biochemistry and Molecular Biophysics, Washington University School of Medicine, Saint Louis, MO 63110

Purification of *S. cerevisiae* mitochondrial DNA polymerase Mip1.

Full-length *Saccharomyces cerevisiae* Mip1 (Pol γ) from yeast S288C (excluding the 29 amino acid N-terminal mitochondrial targeting sequence) was cloned in pET28a and overexpressed in Rosetta cells (DE3) (Novagen). The cells were grown in LB media, induced with 0.5 mM IPTG, and grown overnight at 16 °C. The pellet was resuspended in the lysis buffer A (50 mM Tris-HCl pH 7.5, 600 mM NaCl, 5% (v/v) glycerol 10 mM 2-mercaptoethanol and 1 mM PMSF) and lysed by sonication. The cell lysate was centrifuged at 14,000 rpm for 1 hour at 4°C, and the supernatant was loaded on HisPur Ni-NTA superflow agarose (Thermo Scientific) pre-equilibrated with buffer A. Column was washed with buffer A containing 30 mM imidazole and eluted with buffer A containing 300 mM imidazole. The eluted protein was extensive dialyzed against buffer B (50 mM Tris-HCl pH 7.5, 20% (v/v) glycerol, 2 mM DTT and 0.2 mM PMSF) with 200 mM NaCl. Dialyzed sample was passed through a High Q column (Bio-Rad), collected in the flow-through, loaded on High S column (Bio-Rad), and eluted at buffer A with 700 mM NaCl. The eluted fraction was then diluted in buffer B to reach 150 mM NaCl, and loaded on a heparin column. After washing the column with buffer B with 200 mM NaCl, Mip1 was eluted with buffer B with 700 mM NaCl. After dialysis in Buffer C (20 mM sodium phosphate, pH 7.8, 400 mM NaCl, 10% (v/v) glycerol, 10 mM β-ME and 0.1 mM PMSF), Mip1 was again loaded on HisPur Ni-NTA superflow agarose column, washed with buffer C with 30 mM imidazole and eluted with 300 mM imidazole in Buffer C. Purified Mip1 was dialyzed and stored in storage buffer (50 mM Tris–HCl, pH 8, 600 mM NaCl, 1 mM EDTA, 1 mM DTT and 40% (v/v) glycerol).

Table S1. Sequences of substrates used in the studies. The primer sequence used to generate each primer-template substrate precedes the template sequences used.

Name	Sequence (5’ to 3’)<br>(5’ tail-**G4 or hairpin SEQUENCE**-gap-PRIMER REGION)
R21primer	CY3-CCGCCGCGGAACTTATTAGTG
hTelo	acgtcattggtc**TAGGGTTAGGGTTAGGGTTAGGG**tttttCACTAATAAGTTCCGCGGCGG-Bio
cMyc_ shortgap	acgtcattggtc**TGGGGAGGGTGGGGAGGGTGGGGAAGG**ttttCACTAATAAGTTCCGCGGCGG-Bio
cMyc_ longgap	acgtcattggtc**TGGGGAGGGTGGGGAGGGTGGGGAAGG**ttttcgacaagtacatgattCACTAATAAGTTCCGCGGCGG-Bio
G3T7	acgtcattggtc**GGGTTTTTTTGGGTTTTTTTGGGTTTTTTTGGG**ttttCACTAATAAGTTCCGCGGCGG-Bio
G3T5	acgtcattggtc**GGGTTTTTGGGTTTTTGGGTTTTTGGG**ttttCACTAATAAGTTCCGCGGCGG-Bio
G3T3	acgtcattggtc**GGGTTTGGGTTTGGGTTTGGG**ttttCACTAATAAGTTCCGCGGCGG-Bio
G3T1	acgtcattggtc**GGGTGGGTGGGTGGG**ttttCACTAATAAGTTCCGCGGCGG-Bio
G4T12	**GGGGTTTTTTTTTTTTGGGGTTTTTTTTTTTTGGGGTTTTTTTTTTTTGGGG**ttttCACTAATAAGTTCCGCGGCGG-Bio
G4T6	acgtcattggtc**GGGGTTTTTTGGGGTTTTTTGGGGTTTTTTGGGG**ttttCACTAATAAGTTCCGCGGCGG-Bio
G4T4	acgtcattggtc**GGGGTTTTGGGGTTTTGGGGTTTTGGGG**ttttCACTAATAAGTTCCGCGGCGG-Bio
G4T2	acgtcattggtc**GGGGTTGGGGTTGGGGTTGGGG**ttttCACTAATAAGTTCCGCGGCGG-Bio
Bioprimer	BioTEG/T/Cy3i/ TGGAATTCGAGCTCGGTACCC
cMyc_ Bioprimer	acgtcattggtc**TGGGGAGGGTGGGGAGGGTGGGGAAGG**ttttGGGTACCGAGCTCGAATTCCA
M13hp	acgtcattggtc**GAATTCCCCGGATCCGTCGACCTGCAGGTCGACGGATCCGGGGAATTC**ttttcgacaagtacatgatt GGGTACCGAGCTCGAATTCCA
Chr7hp	aaatccatggaag**CCGCCCAATGTCGCGGGttgaCCCGCGACATTGGGCGG**aaagcttcgta GGGTACCGAGCTCGAATTCCA
nostruc	aaatttgatatgcgacattgggcggaaagcttcgtaGGGTACCGAGCTCGAATTCCA

Figure S1. A) Native PAGE of the G-forming sequences annealed to the Cy3-labeled primer (100 mM KCl, 8 mM $MgAc_2$). $(G_4T_4)_3G_4$ shows an unassigned faster migrating species; however, all $(G_4T_x)_3G_4$, including $(G_4T_4)_3G_4$, blocked DNA synthesis (Fig. 2 and Fig. S5B). B) CD scans for cMyc and hTelo G4s in 100 mM KCl and 8 mM $MgAc_2$. Scans are an average of 5 runs. C) UV Melting curves for hTelo at different salt concentrations. The values of T_m are noted in each graph. D) UV melting curves similar to B, but with the addition of 8 mM $MgAc_2$. E) UV melting curves similar to (A) for cMyc comparing melting at 10 mM KCl with and without the presence of 8 mM $MgAc_2$.

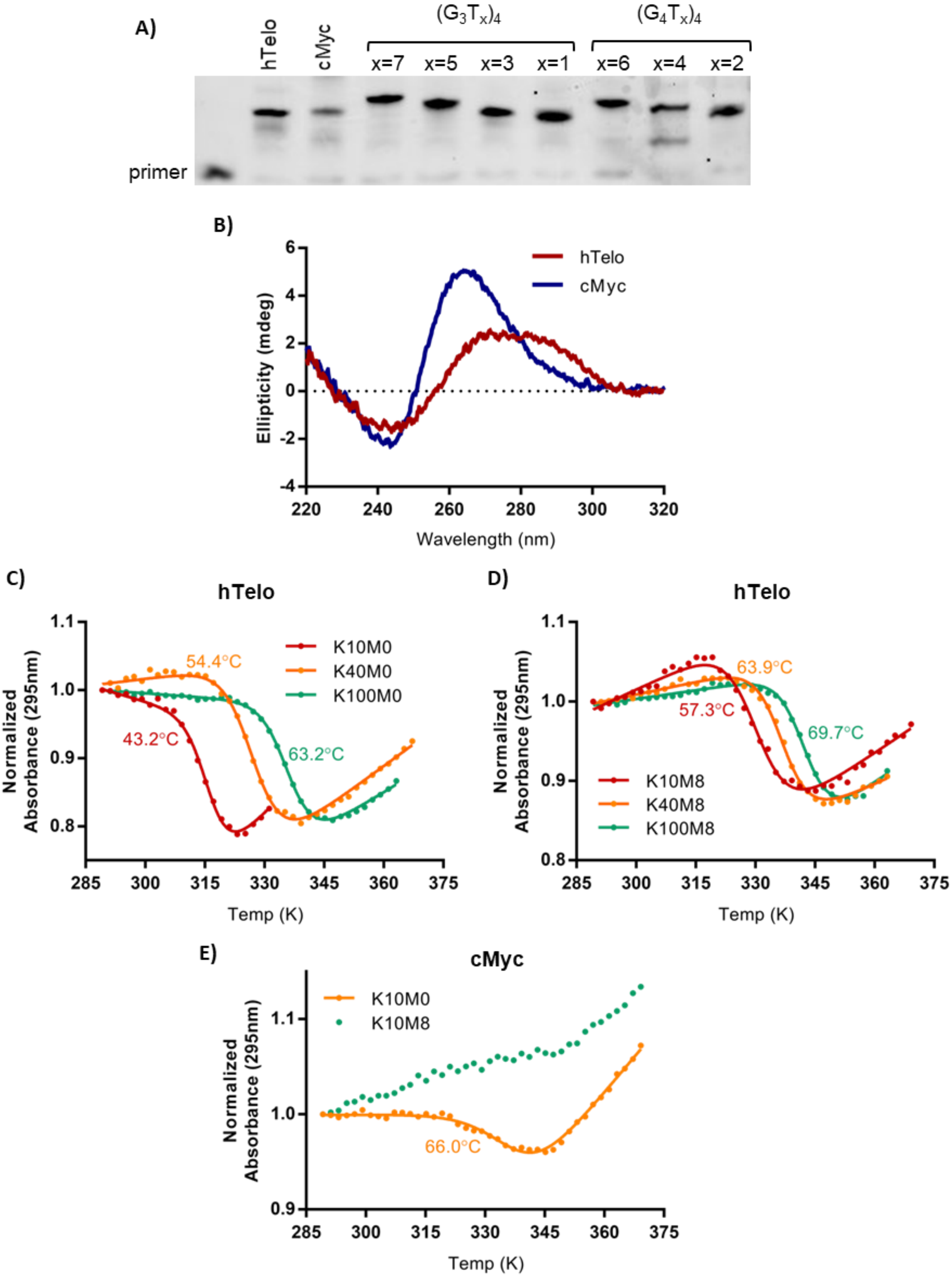

Figure S2. Representative primer extension assays performed with Pol δ and cMyc (left) or hTelo (right) in the template strand.

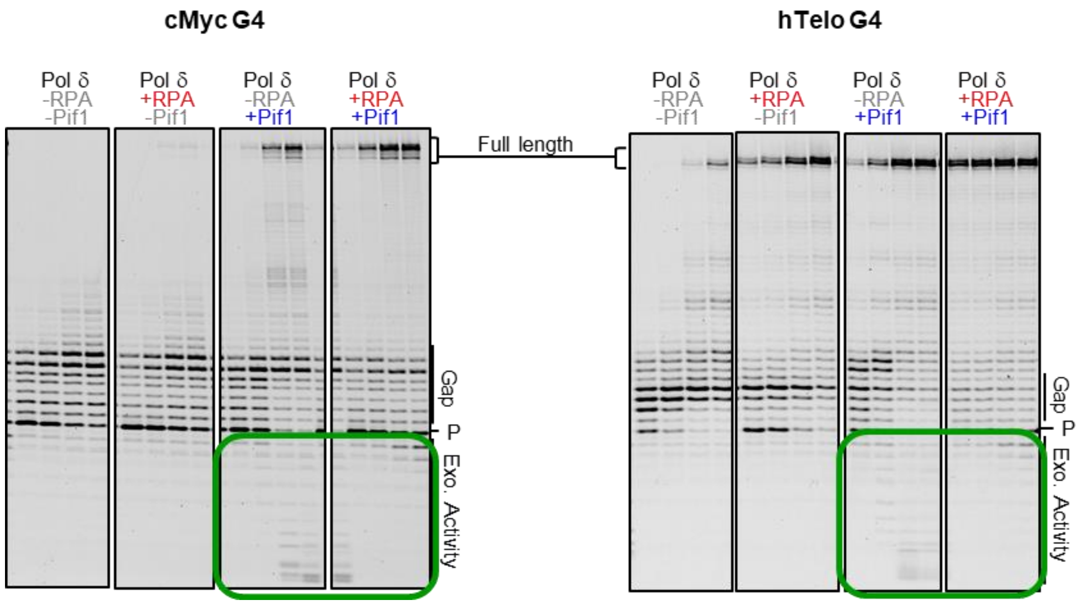

Figure S3. A) Pol δ stalling is dependent on stability of formed G4. Quantification of full-length product formed by Pol δ^{DV} on specified substrate in the presence of Na^+ or K^+. B) Pif1 stimulation of DNA synthesis through a G4 is dependent on helicase activity. Primer extension assays performed with a helicase dead version of Pif1 (Pif1Δ K264A) at 4x or 16x excess. C) (left) Representative primer extension assays on DNA substrate containing cMyc G4 and a 20 nt gap. (right) Quantification of primer extension assays with 3 replicates.

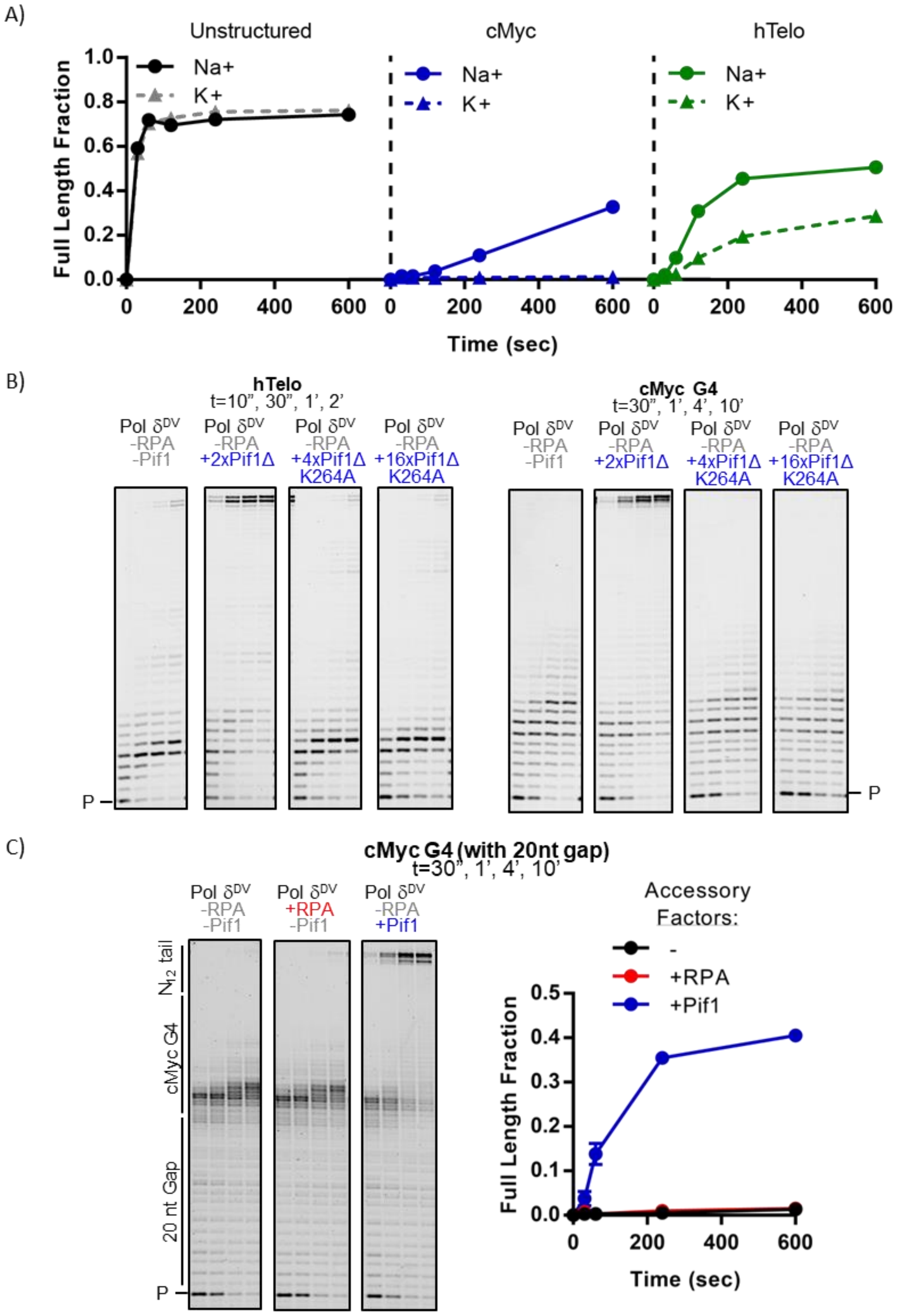

Figure S4. CD spectra (left), in 100 mM KCl and 8 mM $MgAc_2$, and UV melting curves (right) for 3G-stack G4s (A) or 4G-stack G4s (B). Normalized absorbance at 295 nm was fitted to a two-state model. Calculated melting temperature is noted in graph with the corresponding color. C) Quantification of the fraction of full-length product formed at 10 min by Pol δ and Pol δ^{DV} for each G4 (n=3).

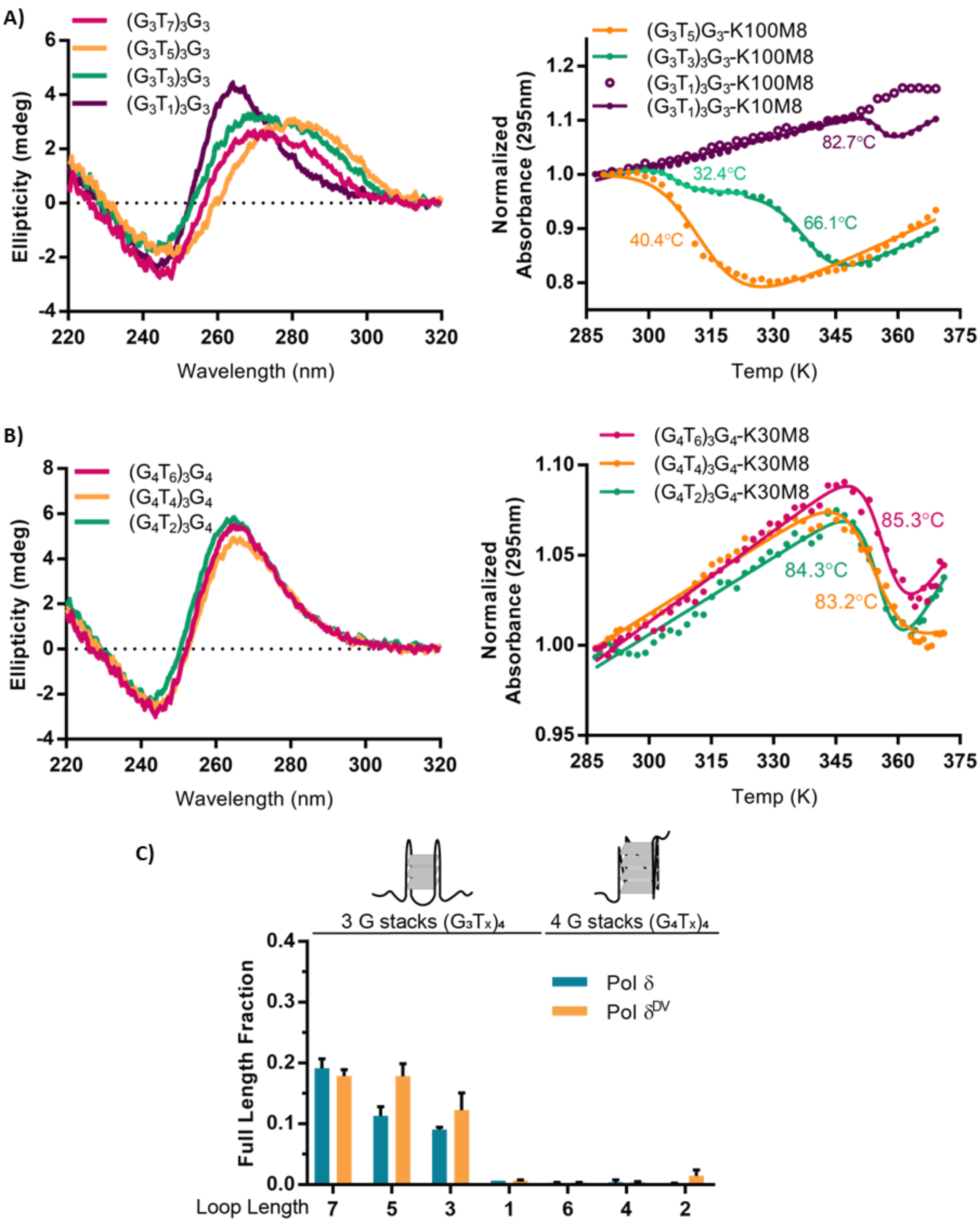

Figure S5. Representative primer extension assay (times: 30”, 1’, 4’, 10’) with Pol δ^{DV} and the 3G-stack G4s (A) or 4G-stack G4s (B) in the presence of Pif1. C) Quantifications of fraction of full-length product generated at 10 min by Pol δ^{DV} with and without Pif1, comparing DNA substrates with and without a 5’ tail. (n=3)

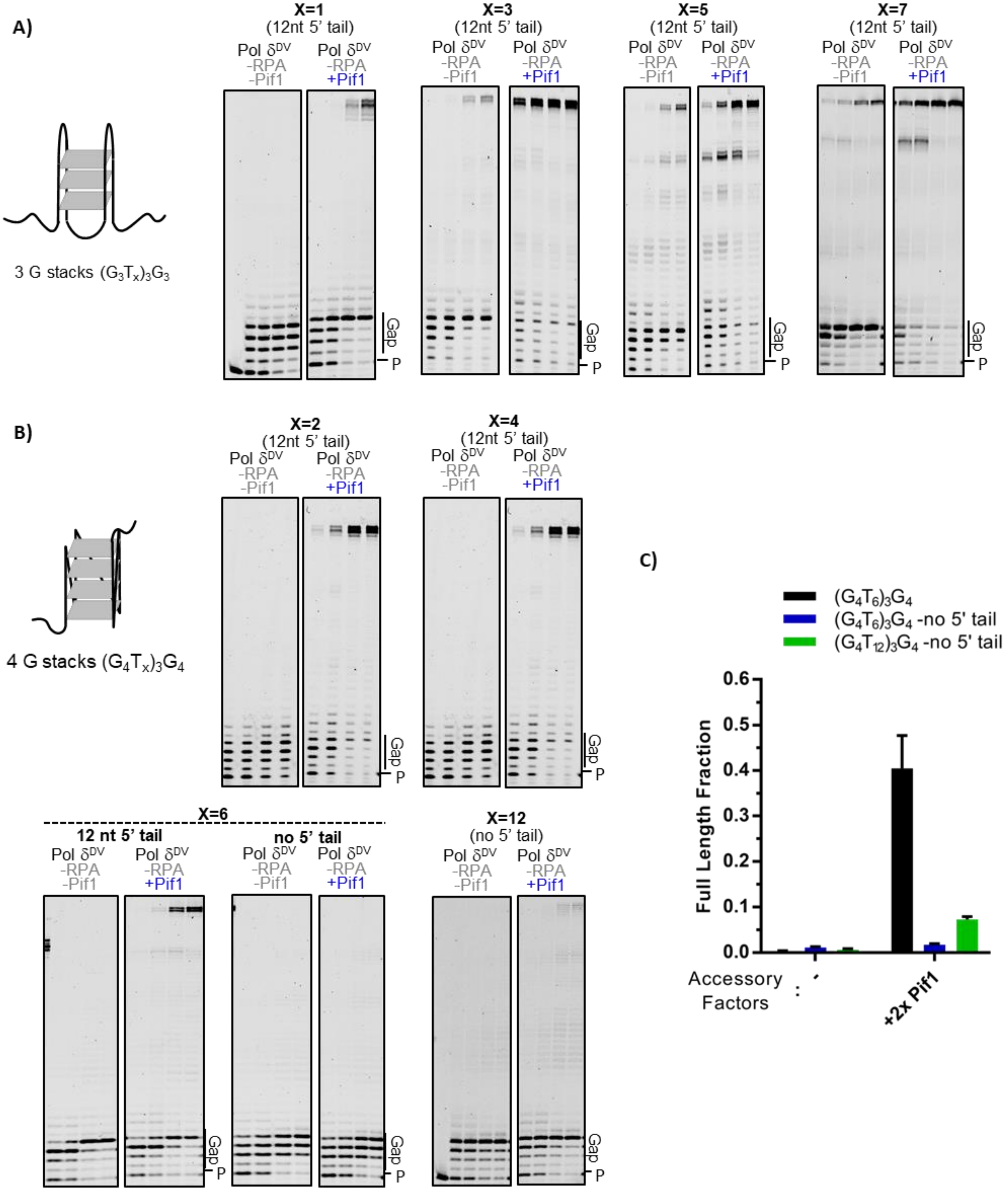

Figure S6. Representative primer extension assay (times: 30”, 1’, 4’, 10’) with Pol δ and the 3G-stack G4s (A) or 4G-stack G4s (B) with or without the presence of RPA.

A)

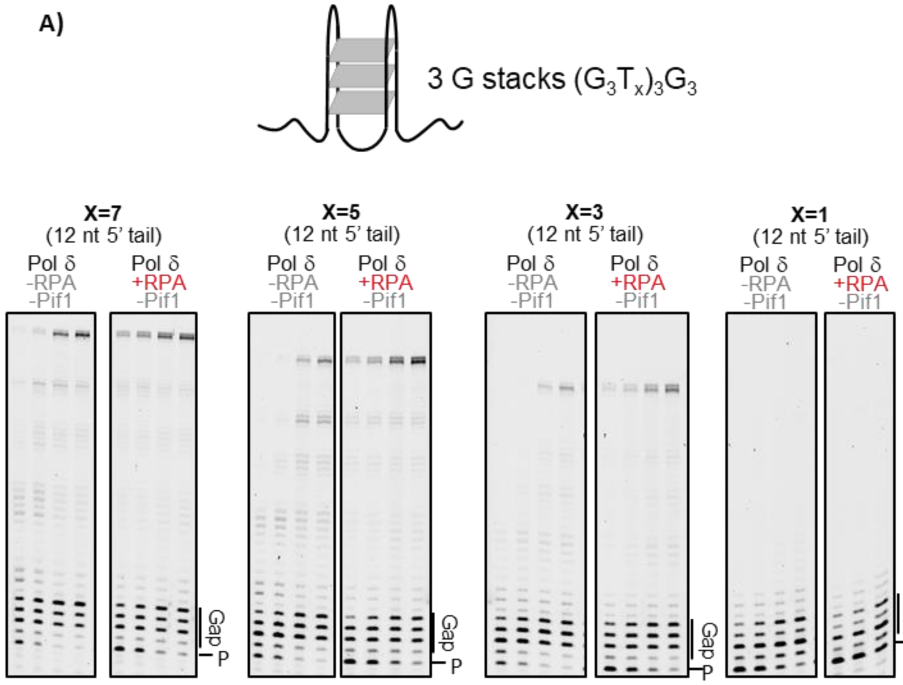

B)

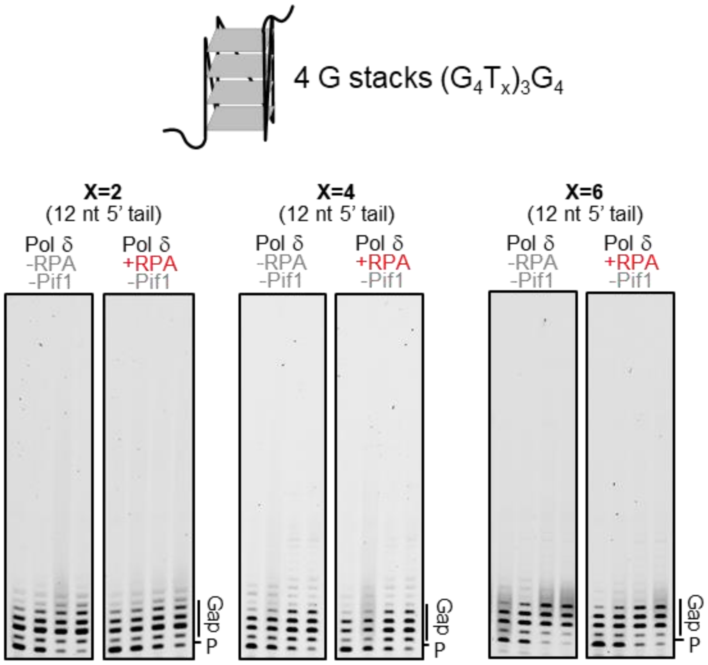

Figure S7. A) Representative primer extension assay (times: 30", 1', 4', 10') with Pol δ^{DV} and the 3G-stack G4s (A) or 4G-stack G4s (B) in the presence of large excess RPA. C**)** Quantifications of full-length product generated at 10 min (n=3).

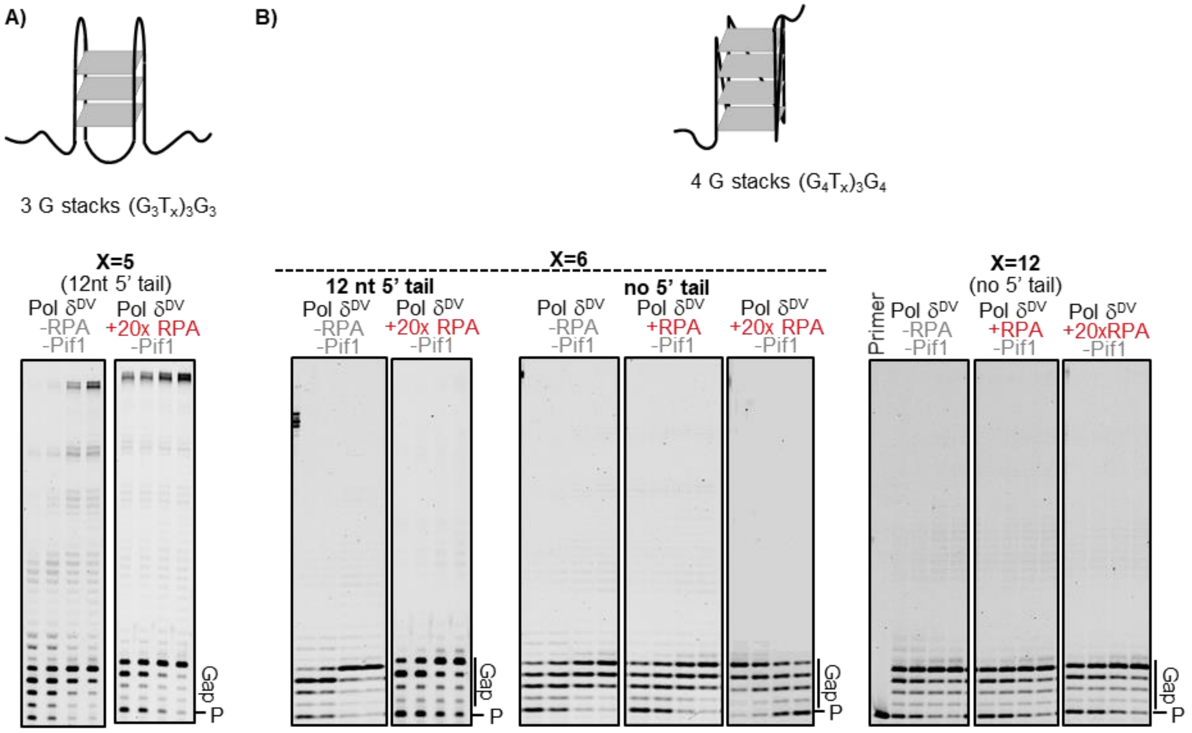

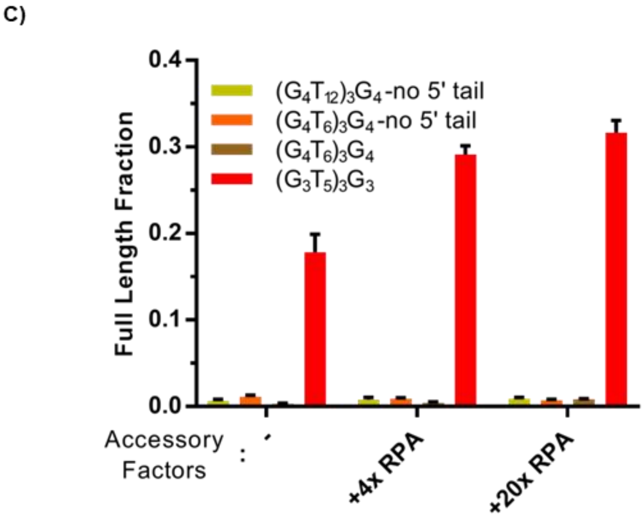

Figure S8. Representative primer extension assays (times: 30”, 1’, 4’, 10’) for two hairpin substrates replicated by Pol δ in the presence of RPA, Pif1, or both.

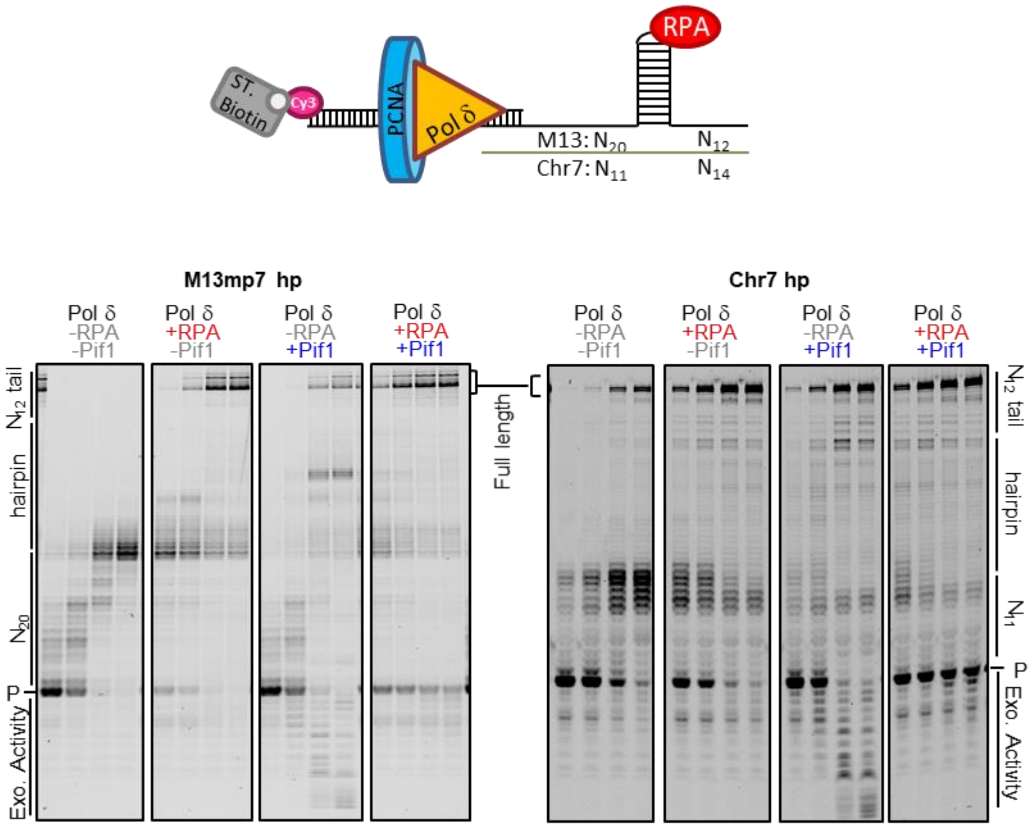

Figure S9. Purified Mip1 is active for exonuclease activity (left) and strand displacement DNA synthesis (right).

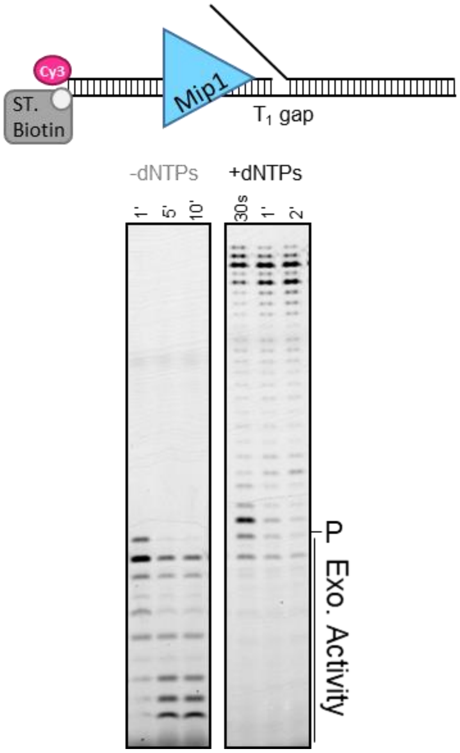

Figure S10. A) Representative primer extension assay (times: 30”, 1’, 4’, 10’) with Mip1 and the specified G4s in the presence of 40x Rim1 (relative to the DNA concentration) or 4x hmtSSB with or without 2x Pif1. B) Quantification of the fraction of full-length product of cMyc (left) and hTelo (right) by Mip1 as a function of time in the presence of Rim1 or hmtSSB with and without the presence of Pif1 (n=3).

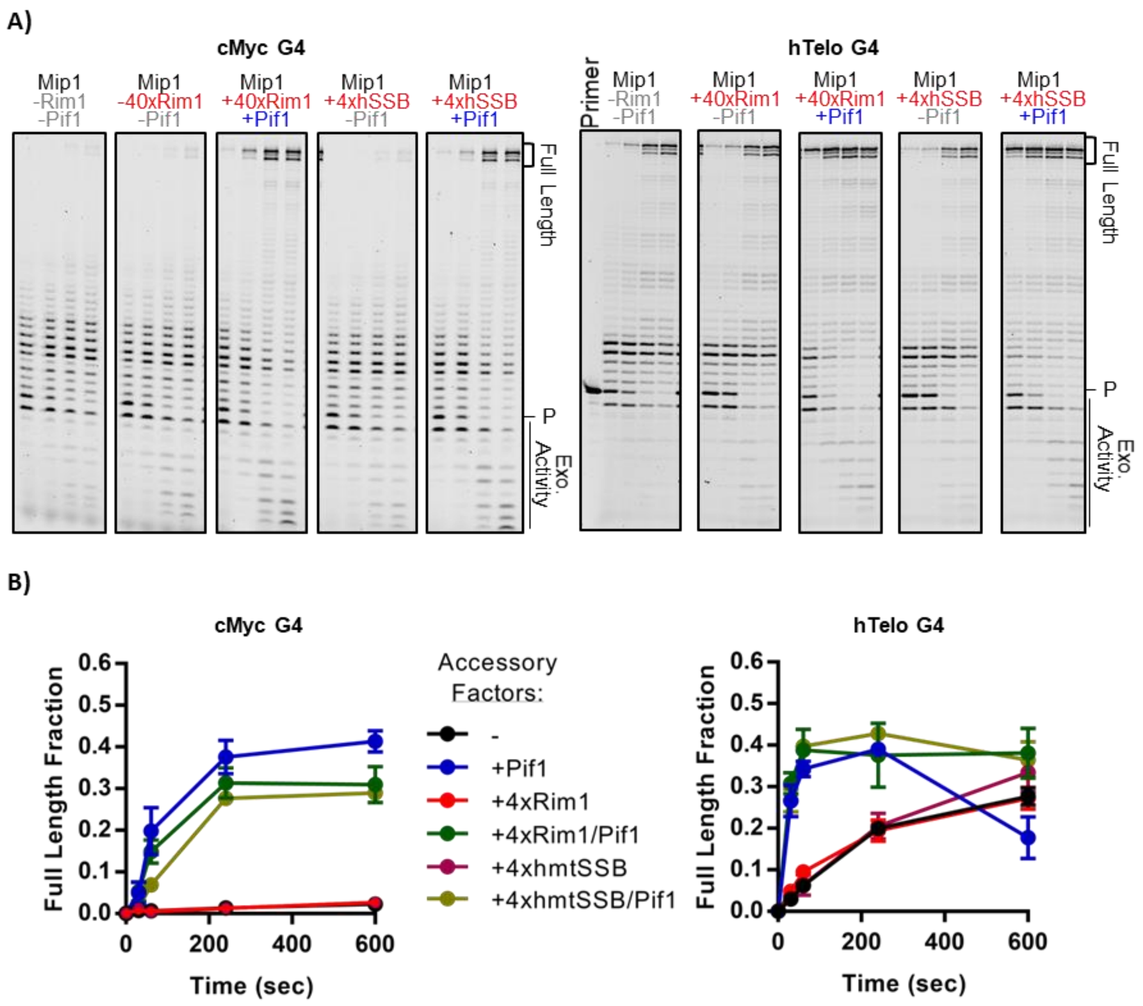

Figure S11. A) Representative primer extension assay (times: 30”, 1’, 4’, 10’) by Mip1 and the specified hairpins (M13mp7 and Chr7) in the presence of Rim1, Pif1, or both. B) Quantifications of the full-length product generated (n=3).

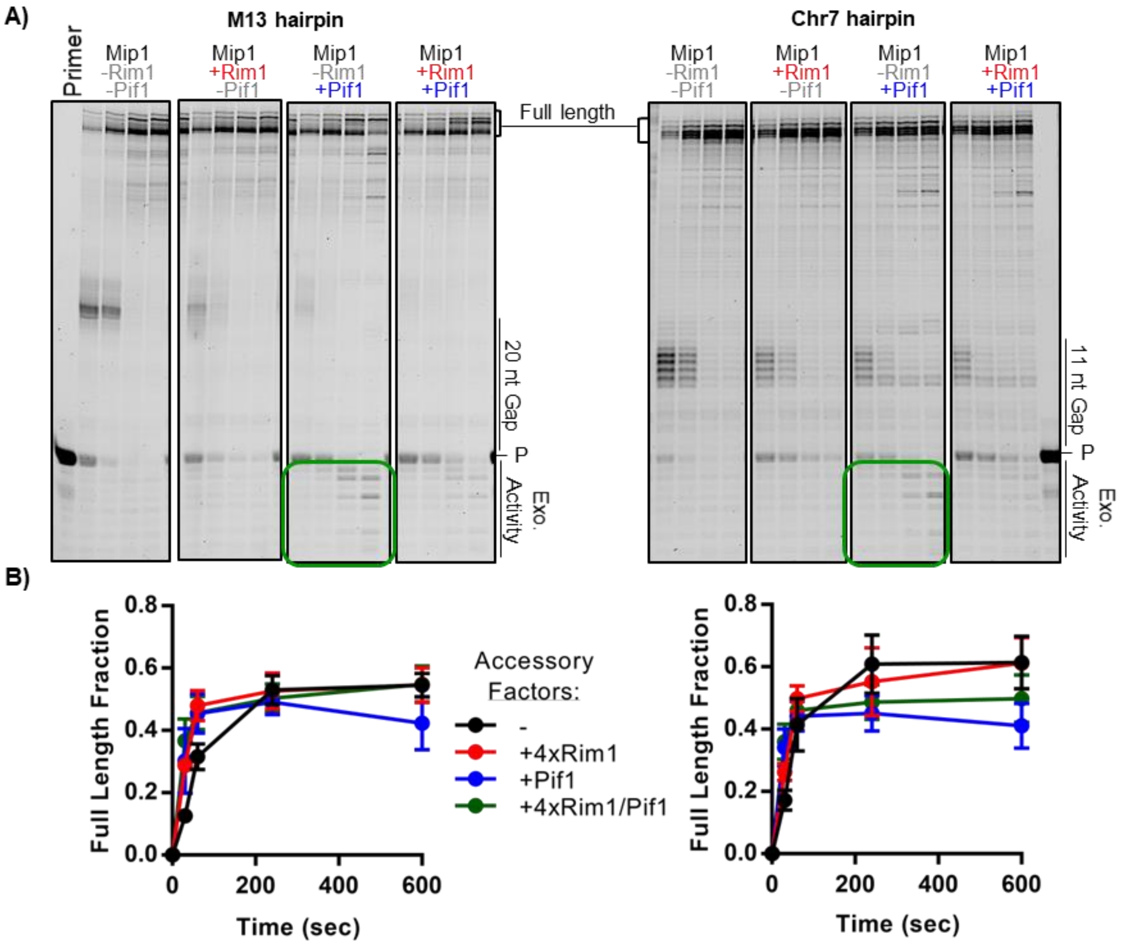

Figure S12. A) Exonuclease activity assay performed under conditions of limited DNA synthesis (only dATP added) using Pol δ with RPA, Pif1, or both on a DNA substrate with cMyc 8 nts away from the 3' end of the primer. B) Similar to A, but on a DNA substrate with the M13mp7 hairpin sequence.

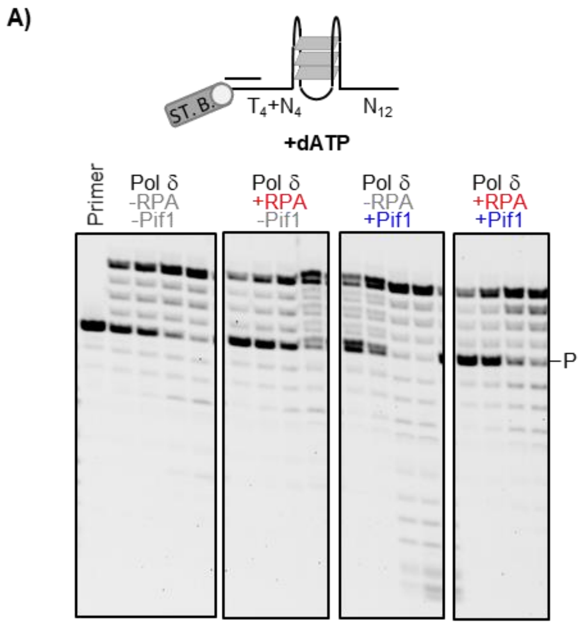

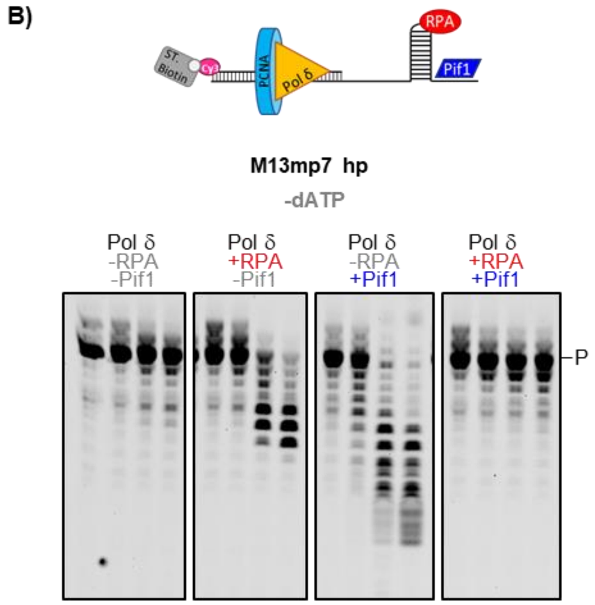

Figure S13. A) Addition of Pif1 alone with or without PCNA or dATP does not cause primer degradation. Time points are taken at 10 min. B) Pol δ incubated with free primer (no template strand) for 1 min. C) Pre-incubation of Pif1 does not result in quicker primer degradation following the addition of Pol δ for 1 min. PCNA was loaded on DNA primer template substrate and then Pif1 was added to the reaction for 0, 1', 4', or 10'. Then Pol δ was added for 1 min and extent of exonuclease activity was measured. D) Exonuclease activity assay performed in the absence of dNTPs using Pol δ with RPA, Pif1, or both on DNA substrates with cMyc with biotinylation on either the top or bottom strand.

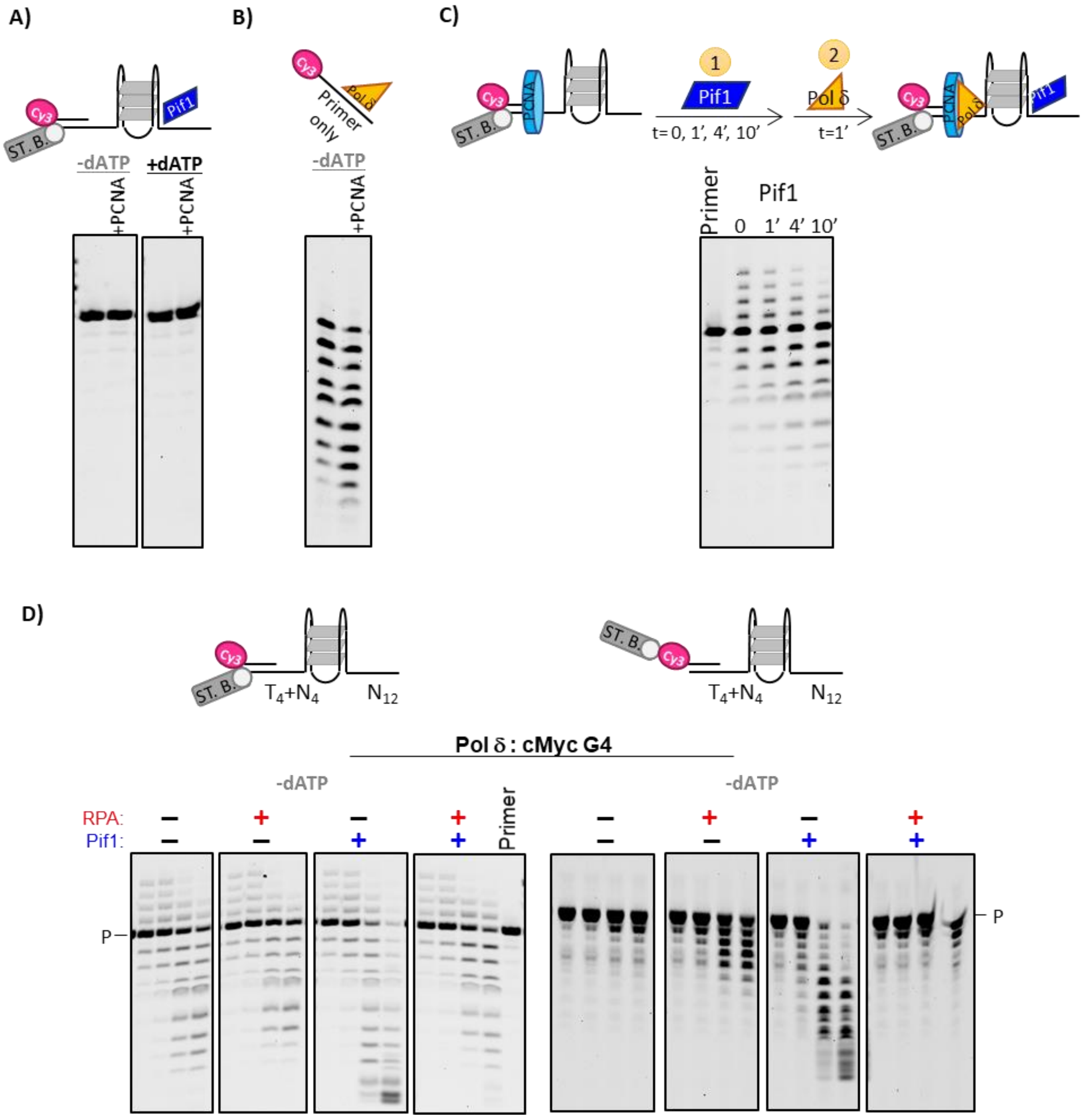

Figure S14. Quantification of excessive primer degradation stimulated by Pif1 shown in Figure 4.

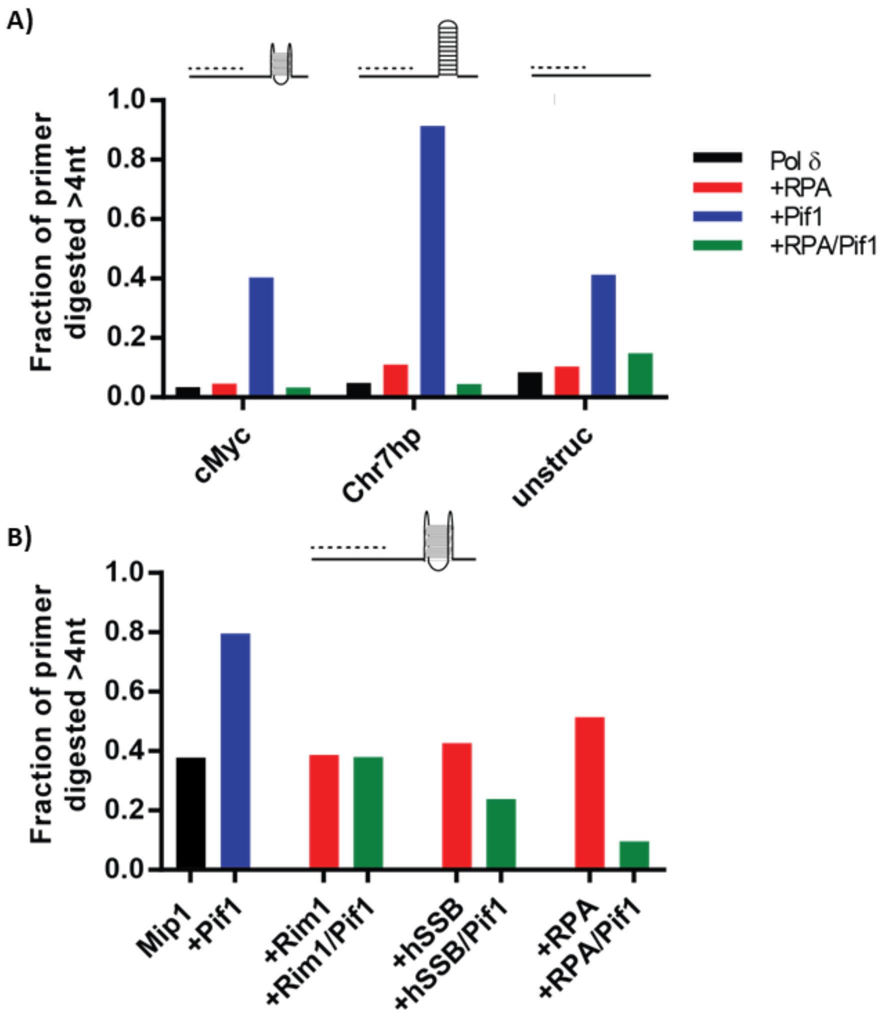

Supplement: gkz608_Supplemental_File [file gkz608_supplemental_file.pdf]
